# Diverse physiological roles of the MqsR/MqsA toxin/antitoxin system

**DOI:** 10.1093/sumbio/qvae006

**Published:** 2024-04-22

**Authors:** Viviana Sanchez-Torres, Joy Kirigo, Thomas K Wood

**Affiliations:** Escuela de Ingeniería Química, Universidad Industrial de Santander, Bucaramanga 680002, Colombia; Department of Chemical Engineering, Pennsylvania State University, University Park, PA 16802, United States; Department of Chemical Engineering, Pennsylvania State University, University Park, PA 16802, United States

**Keywords:** phage defense, bile stress, oxidative stress response, persistence

## Abstract

There is tremendous interest in the use of bacteriophages (phages) to combat multi-drug resistant bacteria. However, to implement successfully phage therapy, host defense systems must be understood. Toxin/antitoxins (TAs) are the most prevalent phage defense system, and the MqsR/MqsA TA system is one of the best-studied systems. This phage-defense system was discovered in a whole-cell, population-averaged, transcriptome study designed to elucidate the biofilm-related genes of *Escherichia coli* in 2004. Biofilms are cells cemented to themselves or to surfaces. Since its characterization (as of April 2024), MqsR/MqsA has been utilized in over 1200 manuscripts, although its role in cell physiology has been contested. Here, we summarize the important physiological roles of this TA system, including its role in (i) the general stress response via repression of *rpoS*, (ii) biofilm formation via repression of *csgA*, (iii) combating bile acid stress in the gastrointestinal tract by inhibiting uptake of the bile salt deoxycholate, (iv) oxidative stress based on single-cell transcriptome studies, and (v) phage defense leading to the persister state.

**Sustainability Statement**

This review is most-related to the sustainable goal of “Good health and wellbeing” in that bacterial infection is likely to be the chief cause of death by 2050 ($100 trillion cost, 10 million deaths/yr) (Udaondo and Matilla 2020), and phages are currently one of the best resources to combat multi-drug resistant bacteria. However, to implement successfully phage therapy, host defense systems against phages must be understood, and TAs are the most prevalent phage defense system. Hence, it is germane to understand the role of TAs in cell physiology.

## Review

### Discovery of MqsR/MqsA

Toxin/antitoxins (TAs) consist of neighboring genes that encode a toxin, which inhibits cell growth, and an antitoxin that directly or indirectly masks the toxin activity (Wang et al. 2021). Based on antitoxin activity at the RNA or protein level, TAs are classified into either seven (Wang et al. 2021) or eight groups (types) (Song and Wood 2020a, 2020b). Critically, TAs are present in almost all bacterial and archaeal species (Yamaguchi et al. 2011, Wang et al. 2021), and numerous distinct systems are often found in the same strain. For example, *Escherichia coli* has at least 39 TAs found to date (Song and Wood 2020b). The primary physiological roles of TAs include phage defense (Pecota and Wood 1996, Hazan and Engelberg-Kulka 2004, Fineran et al. 2009), plasmid stabilization (Ogura and Hiraga 1983), mobile genetic element stabilization (Wozniak and Waldor 2009, Soutourina 2019), plasmid copy number control (Ni et al. 2021), and biofilm formation (Ren et al. 2004, Kim et al. 2009).

Using one of the first whole-population transcriptome studies for biofilms, the *E. coli* genes *mqsRA* (formerly the uncharacterized *ygiUT* locus) were identified as highly induced (Ren et al. 2004). This study also led to the discovery of both the TA system Hha/TomB (Marimon et al. 2016) and the AI-2 transporter TqsA (Herzberg et al. 2006), as well as linked for the first time TAs to biofilms. MqsR/MqsA was recognized as a TA system by determining their X-ray crystallographic structure (Brown et al. 2009), where it was discovered toxin MqsR is an RNase, and antitoxin MqsA is highly structured in the absence of the toxin and binds zinc. The MqsR mRNA cleavage site was determined to be 5′-GCU (Yamaguchi et al. 2009), and MqsR was found to be active only on single-stranded RNA, but cleaves mRNA with 5′-triphosphate, 5′-monophosphate, and 5′-hydroxyl groups (Chowdhury et al. 2016b). Moreover, the crystal structure of MqsA with its promoter showed MqsA bends DNA by over 55° to obtain symmetrical DNA binding (Brown et al. 2011), and the binding of toxin MqsR inhibits DNA-binding by MqsA (Brown et al. 2013).

### MqsA represses the general stress response and curli/biofilm formation

**Figure 1.**
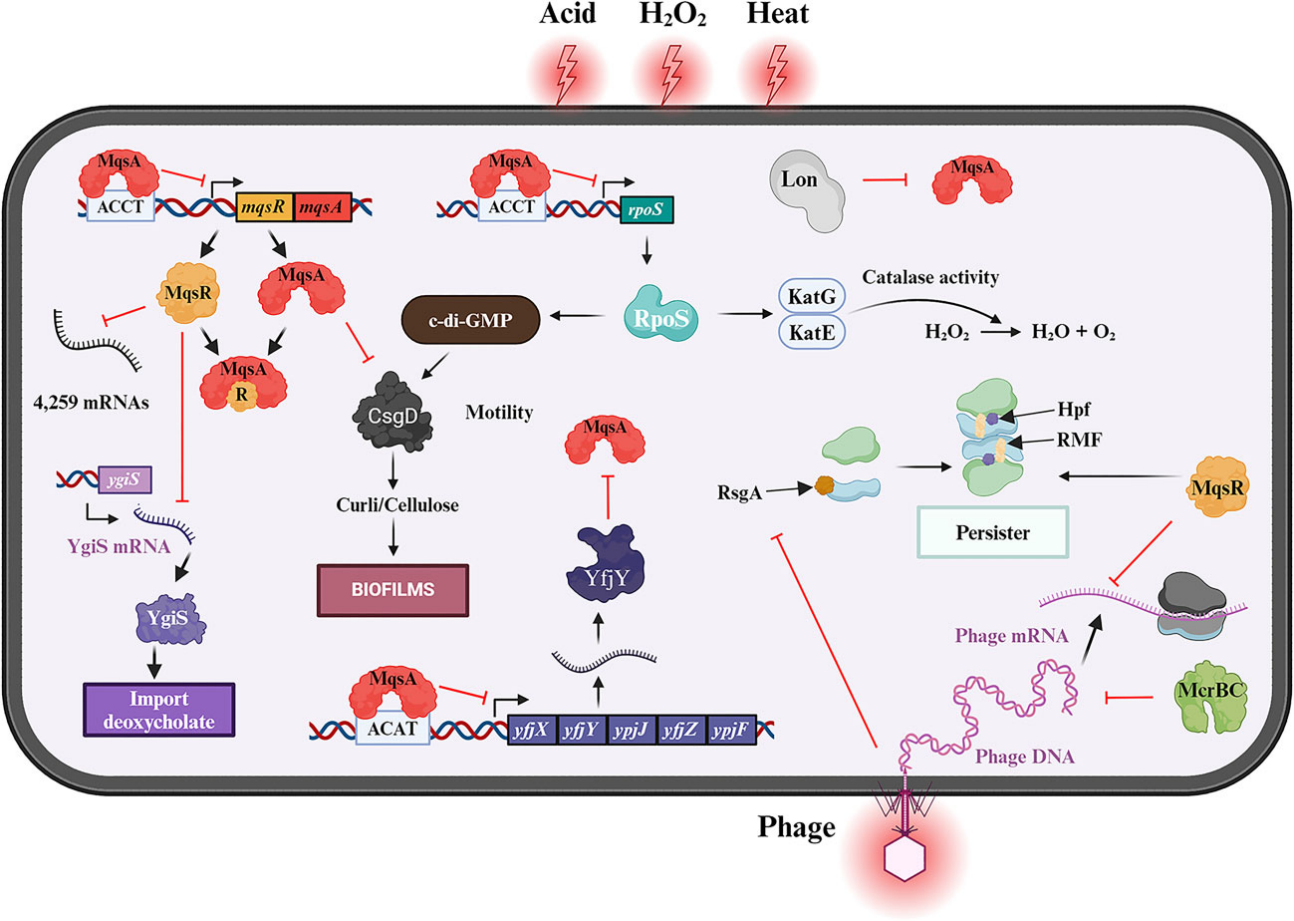
Schematic showing that the myriad roles of the MqsR/MqsA toxin/antitoxin system in cell physiology including in (i) the general stress response via MqsA repression of the *rpoS* promoter by binding at the palindrome 5′-ACCT TGC AGGT (note only the first part of the palindrome is shown in the figure due to space limitations), (ii) biofilm formation via MqsA repression of the *csgD* promoter by binding at the palindrome 5′-ACCT TA AGGT, (iii) combating bile acid stress in the gastrointestinal tract by inhibiting uptake of the bile salt deoxycholate via MqsR degradation of YgiS mRNA (YgiS imports bile acid), (iv) oxidative stress based on single-cell transcriptome studies that identified YfjY protease inhibition of MqsA and MqsA binding of the *yfj* operon near palindrome 5′-ACAT TA ACAT, and (v) phage defense leading to the persister state based on MqsR and McrBC degradation of phage mRNA as monitors of transcription. Created with BioRender.com.

After its initial link to biofilm formation, the MqsR/MqsA TA system was found to impact the general stress response through the regulation of the alternative sigma factor RpoS (Wang et al. 2011); RpoS is the central regulator of the response of the cell to diverse stresses, including nutritive, oxidative, pH, cold/heat shock, osmolarity, and DNA damage stresses (Bouillet et al. 2024). By binding the *rpoS* promoter (Fig. 1) via its conserved palindrome (5′-ACCT TGC AGGT for *rpoS* vs. 5′-ACCT TTT AGGT for *mqsRA*), MqsA represses oxidative stress resistance by reducing catalase activity (Wang et al. 2011); catalase converts hydrogen peroxide into water and oxygen. Furthermore, MqsA reduces cyclic diguanylate thereby increasing motility and reducing biofilm formation (Wang et al. 2011). Hence, during stress, MqsA is degraded by Lon protease, RpoS is derepressed, and both catalase activity and biofilm formation are increased (Wang et al. 2011).

In a similar manner, by binding another palindromic area in the *csgD* promoter (5′-ACCT TA AGGT), MqsA reduces biofilm formation by reducing curli (surface amyloid fibers) and cellulose formation (Soo and Wood 2013) (Fig. 1). CsgD is the master regulator of *E. coli* biofilm formation as it positively regulates both operons that encode proteins for curli synthesis and secretion, *csgDEFG* and *csgBAC*, as well as positively regulates cellulose synthesis (Soo and Wood 2013). Hence, through its influence on the stress response and biofilm formation, MqsA was the first antitoxin shown to be a global regulator; i.e. the first antitoxin shown to regulate more than its own locus by binding at palindromic locations at other positions on the chromosome (Wang et al. 2011, Soo and Wood 2013).

Notably, the TisB/IstR1 TA system has been recently found to mediate the SOS stress response (due to double-strand breaks in DNA) (Cayron et al. 2024); hence, TAs have been further linked to the stress response. Moreover, TAs have been further linked to *Bacillus subtilis* biofilms through TAs TxpA/RatA and YqcG/YqcF (Bloom-Ackermann et al. 2016, Kobayashi 2021).

### MqsR/MqsA control the GhoT/GhoS TA system

In an additional regulatory role, the MqsR/MqsA TA system also controls the GhoT/GhoS TA system since toxin MqsR degrades preferentially the mRNA of antitoxin GhoS, which contains three 5′-GCU cleavage sites, whereas the toxin GhoT mRNA lacks cleavage sites (Wang et al. 2013). This selective, global mechanism of specific mRNA degradation by MqsR was termed “differential mRNA decay” (Wang et al. 2013) and led to the discovery of the GhoT/GhoS type V TA system (Wang et al. 2012), since GhoT mRNA is one of only 14 *E. coli* transcripts that lack a MqsR 5′-GCU cleavage site. Therefore, during stress, toxin GhoT mRNA is enriched (Wang et al. 2013), toxin GhoT is formed, and GhoT damages the cell membrane leading to reduced ATP levels and reduced growth (Cheng et al. 2014). Hence, MqsR/MqsA is the first TA system shown to control another TA system as a cascade.

### MqsR/MqsA reduces bile acid stress

Along with roles in regulating the general stress response (which includes oxidative stress) and in controlling other TAs, the MqsR/MqsA TA system is involved in the regulation of bile acid stress (Fig. 1), another form of oxidative stress. Critically, an unmarked deletion of *mqsRA* causes higher metabolic activity in the presence of 4.5% deoxycholate (a prevalent bile-acid derivative in the gastrointestinal tract) but reduces growth compared to the wild-type strain (Kwan et al. 2015). The mechanism by which MqsR/MqsA improves growth of the wild-type strain in bile acid is that MqsR reduces production of YgiS through cleavage of YgiS mRNA, which contains 43 MqsR cleavage sites (Kwan et al. 2015). Prior to this work, YgiS was an uncharacterized, predicted periplasmic binding protein, and it was found that YgiS imports deoxycholate (Kwan et al. 2015). Hence, MqsR increases fitness in the presence of bile acid during the passage of *E. coli* in the gastrointestinal tract by reducing its exposure to this oxidizing agent by reducing YgiS. These results are important since it is rare to find a phenotype with TAs upon deletion of the TAs due to redundancy of TAs in most bacteria (Verstraeten et al. 2015), and many TAs reports rely on overproduction of the toxin (Fraikin et al. 2020).

### Dispute regarding the physiological role of MqsR/MqsA

Despite these convincing results of the roles of MqsR/MqsA in cell physiology, the Van Melderen group published a report based on their negative results that claimed there was no induction of *mqsRA* and no phenotype upon deleting *mqsRA* during stress (Fraikin et al. 2019). However, within a handful of months, the Van Melderen publication was undermined by the Laub group, which found *mqsRA* is induced dramatically both during amino acid stress (181-fold) and during oxidative stress (90-fold) (LeRoux et al. 2020). Unfortunately, the Laub group also reported that it was unable to find a phenotype for MqsR/MqsA during stress (LeRoux et al. 2020), stating “TAs are unlikely to be critical or conserved components of bacterial stress response systems”; however, this group did not investigate bile acid stress, phage defense, or biofilm phenotypes. Furthermore, their results were highly suspect as they used a TA system deletion strain that has many non-related mutations (e.g. large chromosomal inversions) (Goormaghtigh et al. 2018); the use of TA system deletion strains with non-intended mutations has notoriously led to three retractions, based on the errors we have described (Wood and Song 2020). Critically, within 2 years, the Laub group (LeRoux et al. 2020) contradicted itself twice in that they reported results where toxins of TAs were active by showing the DarT/DarG TA system defends against RB69 and T5 phages (LeRoux et al. 2022) and by showing that the MqsR/MqsA/MqsC TA system indeed has a phenotype in that it also defends against T2 phage (Vassallo et al. 2022). Moreover, the Laub group now recognizes TAs primarily are used for phage defense (LeRoux and Laub 2022), as first shown a quarter of a century earlier for Hok/Sok (Pecota and Wood 1996), and confirmed thereafter for MazF/MazE (Hazan and Engelberg-Kulka 2004) and ToxN/ToxI (Fineran et al. 2009).

### MqsR/MqsA and the oxidative stress response through single-cell transcriptomics

We predicted in 2022 that single-cell approaches would lead to insights into the role of TAs in cell physiology (Wood 2022). Therefore, by using this technique (Fernández-García et al. 2024b), we hoped we could provide additional evidence regarding the physiological role of MqsR/MqsA and address the controversy surrounding it. We focused on oxidative stress since this is an almost universal stress for procaryotes growing both aerobically and anaerobically (Khademian and Imlay 2021), and MqsA is part of the regulation machinery for the RpoS-controlled oxidative stress response (Wang et al. 2011) and part of the bile acid stress response, which resembles oxidative stress (Kwan et al. 2015). We also used a strain with a deletion of the *mqsRA* locus without antibiotic resistance genes (Kim et al. 2010).

We found that upon oxidative stress (20 mM H_2_O_2_ for 10 min), deletion of *mqsRA* makes the whole population of *E. coli* cells less sensitive; i.e. the wild-type strain has less survival than the *mqsRA* mutant (Fernández-García et al. 2024b). Similar results were found with acid stress (pH 2.5 for 10 min for four cycles), and a deletion of *mqsR* alone (thereby increasing MqsA) makes the *mqsR* mutant more sensitive than the wild-type strain (Fernández-García et al. 2024b). These results are in agreement with the model of MqsA repressing the oxidative-stress response of RpoS.

At the single-cell level, the *mqsRA* mutation affects the transcriptome in a heterogeneous manner during oxidative stress, and in the wild-type strain (containing an intact MqsR/MqsA TA system), the poorly characterized *yfjXY ypjJ yfjZ ypjF* operon of cryptic prophage CP4-57 is induced relative to the *mqsRA* mutant, suggesting this locus confers oxidative stress resistance (Fernández-García et al. 2024b). Corroborating this hypothesis, the *yfjY* deletion causes increased sensitivity to H_2_O_2_, acid, and heat stress, and these phenotypes were complemented. Critically, MqsA controls the *yfjXY ypjJ yfjZ ypjF* operon by binding the operon of the promoter (Fig. 1). Also, YfjY was found to be a protease that degrades MqsA, which results in derepression of *rpoS* and facilitates the stress response. Hence, along with repression of the *rpoS* promoter, the MqsR/MqsA TA system facilitates the stress response through cryptic phage protease YfjY (Fernández-García et al. 2024b). Remarkably, these results demonstrate the host has re-engineered genes of its former foe, phage, for its oxidative stress response (Fernández-García et al. 2024b). Notably, the host must use phage-defense systems like CRISPR-Cas to control the still-lethal genes of its former foe (Song et al. 2022).

### MqsR/MqsA and persistence

The small subpopulation of cells that are formed due to various stresses (e.g. antibiotic, nutritive, oxidative) that survive the stress by becoming dormant, are known as persister cells (Hobby et al. 1942, Johnson and Levin 2013, Wood and Song 2020, Kaushik et al. 2022). MqsR/MqsA of *E. coli* is the first TA system that when the genes that encode it are deleted, persistence is reduced (Kim and Wood 2010); hence, MqsR/MqsA are linked to persistence. These results are significant since almost any toxin protein, when overproduced, increases persistence (Chowdhury et al. 2016a), but few TAs impact persistence when deleted.

The MqsR/MqsA TA system was also used to provide a key insight into the mechanism of persistence: Since production of MqsR RNase increased persistence by 14 000 (Hong et al. 2012), it became clear persistence is induced by ceasing protein production. Similarly, stopping transcription, translation, and ATP production increases persistence 100 000-fold (Kwan et al. 2013). Hence, four methods were discerned for converting the whole population of *E. coli* cells into persister cells (Kwan et al. 2013). These approaches allowed large populations of persister cells to be studied at the single-cell level for the first time (Kim et al. 2018, Song et al. 2019, 2021, Song and Wood 2020c, 2020d; Yamasaki et al. 2020). By using these approaches with single cells, it was found persisters form by inactivating/dimerizing their ribosomes based on the stress alarmone guanosine pentaphosphate/tetraphosphate (henceforth, ppGpp), which increases inactivating factor RaiA and dimerization factors RMF and Hpf; this is known as the “ppGpp Ribosome Dimerization Persister (PRDP)” model (Song and Wood 2020d). Using single-cell microscopy, this approach also led to the discovery that persister cells (i) do not resuscitate spontaneously (Yamasaki et al. 2020) but instead (ii) resuscitate based on the number of active ribosomes (Kim et al. 2018) via ribosome rescue factor HflX (Yamasaki et al. 2020), and (iii) resuscitate when nutrients are recognized through chemotaxis proteins and the phosphotransferase system (Yamasaki et al. 2020). Persister cells wake at different times due to the heterogenous number of active ribosome in resuscitating cells, and upon waking, they grow exponentially in the same manner as the original cells (Kim et al. 2018). Given that all three kingdoms dimerize ribosomes (Beckert et al. 2018, Yaeshima et al. 2022, McLaren et al. 2023, van Schijndel 2023), the PRDP model appears to be a conserved model for how cells survive myriad stresses.

### MqsR/MqsA/MqsC induces persistence during phage defense

The physiological role of phage defense for TAs was discovered in 1996 using the type I Hok/Sok system based on the realization that, like plasmid loss that prevents new antisense Sok RNA formation and leads to the translation of longer-lived Hok toxin mRNA, the cessation of host transcription and translation by phages should also induce TAs (Pecota and Wood 1996). Specifically, it was found that infection by T4 phage, a lytic phage that blocks transcription in 3–4 min, activates Hok toxin through transcription shutoff, which leads to cell membrane damage by Hok that reduces host growth (Pecota and Wood 1996). In effect, the host monitors transcription and reduces metabolism upon phage infection. Since this seminal discovery, the primary physiological role of TAs is now generally considered to be phage inhibition (Song and Wood 2020b, LeRoux and Laub 2022).

The first evidence that phages induce persistence upon was obtained by producing from plasmids all 4287 *E. coli* proteins during phage T4 infection and selecting cells with enhanced survival, then sequencing the plasmids of surviving cells to identify beneficial proteins (Fernández-García et al. 2023). Using this approach, GTPase RsgA was identified, and RsgA appears to inactivate ribosomes and induce persistence to thwart T4 phage (Fernández-García et al. 2023).

Direct evidence that phage infection leads to persistence was obtained using the MqsR/MqsA/MqsC tripartite phage defense system of *E. coli* C496_10 with T2 lytic phage (Fernández-García et al. 2024a). Tripartite TAs use a chaperone component (e.g. MqsC) to facilitate folding and protect the antitoxin from degradation (Bordes et al. 2016). Cells expressing MqsR/MqsA/MqsC inhibited T2 phage by 10^5^-fold and reduced T2 titers by 3000-fold compared to cells that lack the TAs (Fernández-García et al. 2024a), which confirmed that MqsR/MqsA/MqsC is a robust phage-defense system, as reported (Vassallo et al. 2022). Critically, contrary to the first report (Vassallo et al. 2022), six lines of evidence showed cells producing MqsR/MqsA/MqsC do not undergo “abortive infection” (i.e. cell death) during phage infection, but, instead, are in the persister state: (i, ii) tolerance to multiple antibiotics (ciprofloxacin and ampicillin), (iii) heterogeneous resuscitation, (iv) lack of metabolic activity via flow cytometry, (v) persister-cell morphology via TEM, and (vi) kill curves with plateaus similar to antibiotic-induced persisters cells (Fernández-García et al. 2024a). Furthermore, it was discovered that the MqsR/MqsA/MqsC TA system works with the EcoK McrBC restriction-modification system to inactivate T2 phage (Fig. 1). Hence, phage infection, which is arguably the most prevalent stress, induces persistence in a manner similar to other stresses (e.g. antibiotics, starvation, acidity, and oxidation) (Fernández-García et al. 2024a).

In addition, these results refute the idea of “abortive infection” or “programmed cell death” upon phage infection as frequently claimed without convincing evidence (Lopatina et al. 2020, Arias et al. 2022, Johnson and Kranzusch 2022, LeRoux and Laub 2022, LeRoux et al. 2022, Rousset and Sorek 2023). Instead, these results with MqsR/MqsA/MqsC show the host survives phage infection (Fernández-García et al. 2024a). In addition, these results show reliably for the first time that TAs induce the persister state, since the MqsR/MqsA/MqsC phage-defense system was not overproduced artificially using chemical inducers, but, instead, was induced by phage infection (Fernández-García et al. 2024a).

### Other MqsR/MqsA roles

Numerous physiological roles for the MqsR/MqsA TAs have also been reported in non-*E. coli* systems; for example, in *Xylella fastidiosa*, copper stress (Merfa et al. 2016), vesicles (Santiago et al. 2016), and biofilm formation have been linked to the MqsR/MqsA TA system (Lee et al. 2014). In addition, MqsR/MqsA in *Pseudomonas fluorescens* has been reported to play a role in both biofilm formation (Wang et al. 2019) and virulence (Zhang et al. 2023) through the negative regulation of transcription of the gene encoding global regulator AgtR by MqsA. Also, MqsR/MqsA in *Pseudomonas putida* has been reported to play a role in persistence and biofilm formation (Sun et al. 2017).

### Perspectives

This review of the physiological roles of the MqsR/MqsA TA system shows there is considerable evidence that TAs on the whole, and in particular, the MqsR/MqsA and MqsR/MqsA/MqsC TA systems, are robust players in bacterial physiology. Hence, we have moved far beyond wondering “why so many, what for?” (Van Melderen 2010) and beyond hypothesizing that “chromosomally encoded systems might lose their addictive properties, which might be the first sign of degeneration” (Van Melderen 2010) as well as “TAs are progressively lost during evolution” (Fraikin et al. 2020). Furthermore, given their established role in phage defense, this implies that TAs will also be important for sustainable environmental applications since phages are likely to be utilized for more than traditional phage therapy but also for pest control in agriculture, for reducing the spread of antibiotic resistance, and for reducing global warming (García-Cruz et al. 2023).

## Data Availability

All relevant data are contained within this review.
